# Exploring the Role of Genetic and Genomic Factors in Therapeutic Response to Heart Failure: A Comprehensive Analytical Review

**DOI:** 10.3390/genes16070801

**Published:** 2025-07-04

**Authors:** Aurora Ferro, Andrea Segreti, Simone Pasquale Crispino, Riccardo Cricco, Anna Di Cristo, Martina Ciancio, Fiorella Gurrieri, Gian Paolo Ussia, Francesco Grigioni

**Affiliations:** 1 Department of Cardiovascular Sciences, Fondazione Policlinico Universitario Campus Bio-Medico, Via Alvaro del Portillo 200, 00128 Rome, Italy; aurora.ferro@unicampus.it (A.F.); simone.crispino@unicampus.it (S.P.C.); riccardo.cricco@unicampus.it (R.C.); martina.ciancio@unicampus.it (M.C.);; 2 Unit of Cardiovascular Sciences, Department of Medicine and Surgery, Università Campus Bio-Medico di Roma, 00128 Rome, Italy; 3 Department of Biomedical Engineering, Intelligent Health Technology Lab, Università Campus Bio-Medico di Roma, 00128 Rome, Italy; 4 Research Unit of Medical Genetics, Department of Medicine and Surgery, Università Campus Bio-Medico di Roma, 00128 Rome, Italy; 5 Operative Research Unit of Medical Genetics, Fondazione Policlinico Universitario Campus Bio-Medico, 00128 Rome, Italy

**Keywords:** dilated cardiomyopathy (DCM), drug response, epigenetics, gene–environment interaction, gene therapy, genetic variability, guideline-directed medical therapy (GDMT), heart failure (HF), hypertrophic cardiomyopathy (HCM), pharmacogenomics, precision medicine, single-nucleotide polymorphisms (SNPs)

## Abstract

Heart failure (HF) remains a leading cause of morbidity and mortality worldwide. Despite significant advances in pharmacological therapies, responses to treatment vary widely among patients. Growing evidence suggests that genetic factors play a crucial role in influencing individual responses to HF therapies. Genetic variations, including single-nucleotide polymorphisms (SNPs), gene expression profiles, and epigenetic modifications, have been shown to affect drug metabolism, receptor sensitivity, and the molecular pathways involved in HF progression. These genetic determinants may not only predict the efficacy of common therapeutic agents such as angiotensin-converting enzyme inhibitors, beta-blockers, mineralocorticoid receptor antagonists, and sodium-glucose cotransporter-2 inhibitors, but also help identify patients at risk of adverse drug reactions. As personalized medicine continues to advance, a deeper understanding of the genetic basis of drug response in HF could enable more tailored treatment strategies, improving clinical outcomes and minimizing adverse effects. This review explores the current evidence on the genetic underpinnings of response to HF treatment and discusses its potential implications in clinical practice, highlighting current knowledge gaps.

## 1. Introduction

### 1.1. Overview of Heart Failure: Pathophysiology, Etiology, and Classification

Heart failure (HF) is a heterogeneous clinical syndrome characterized by signs and symptoms resulting from structural and/or functional cardiac abnormalities. The diagnosis is typically supported by elevated levels of natriuretic peptides and objective findings indicative of pulmonary and/or systemic congestion [1,2]. It is estimated that HF affects 64 million people globally, and the impact of this global health concern remains significant. Despite therapeutic advances, the condition is still associated with substantial morbidity and a five-year mortality rate of approximately 50%. In developed countries, the prevalence of HF among adults is between 1% and 3%, and this is predicted to rise due to improved diagnostic accuracy and the broader access to life-prolonging treatments after diagnosis [3,4,5]. 

The pathophysiology of HF is characterized by an initial myocardial insult or chronic hemodynamic overload, which activates a range of compensatory neurohormonal and hemodynamic mechanisms (e.g., sympathetic nervous system activation, renin–angiotensin–aldosterone system activation, Frank–Starling, and Bowditch effects). While these initial responses may temporarily support cardiac output, chronic activation can lead to maladaptive responses, characterized by myocyte hypertrophy, increased myocardial oxygen demand, apoptosis, and adverse remodeling. This process initiates a vicious cycle of progressive cardiac deterioration and cell death, ultimately leading to the terminal stages of the disease [6].

Phenotypic heterogeneity reflects etiological diversity, with ischemic heart disease being the most common cause worldwide. Nevertheless, non-ischemic factors must be considered, including long-standing hypertension, valvular heart disease, primary cardiomyopathies, and myocarditis, which also play a significant role [4]. Contemporary guidelines utilize the classification of HF based on left ventricular ejection fraction (LVEF): HF with reduced LVEF (HFrEF, LVEF ≤ 40%), mildly reduced LVEF (HFmrEF, LVEF 41–49%), preserved LVEF (HFpEF, LVEF ≥ 50%), and improved LVEF (HFimpEF, HF with a baseline LVEF ≤ 40%, a ≥10 point increase from baseline LVEF, and a second measurement of LVEF *>* 40%) [1]. Guideline frameworks also delineate the ACC/AHA staging system, which categorizes patients from stages A to D (from at-risk to advanced HF) and employs NYHA functional classes I–IV to stratify symptom severity and guide prognosis and management [2].

Table 1 summarizes the main phenotypes of HF, categorized by LVEF. It provides an overview of their most common underlying causes and the current evidence-based therapeutic recommendations [1,4,7].

### 1.2. Current Therapeutic Landscape: From Pharmacology to Precision Medicine

The goals of pharmacological treatment for HF are to relieve symptoms, reduce mortality, and improve quality of life. The current “four-pillar therapy” for HF includes ACE inhibitors (ACEi) or angiotensin receptor-neprilysin inhibitors (ARNI), beta-blockers, mineralocorticoid receptor antagonists (MRAs), and sodium-glucose cotransporter-2 inhibitors (SGLT2i). These drugs target the Renin-Angiotensin-Aldosterone System (RAAS) and the sympathetic nervous system, while SGLT2i (dapagliflozin or empagliflozin) is recommended regardless of ejection fraction to reduce hospitalization and mortality. Loop diuretics (e.g., furosemide) are employed for the management of fluid retention. Ivabradine and digoxin may be considered in patients with HFrEF who remain symptomatic despite optimized therapy [1]. Vericiguat, a soluble guanylate cyclase stimulator, may be added in HFrEF patients with recent decompensation despite standard therapy [8].

Non-pharmacological strategies include exercise-based cardiac rehabilitation and multi-disciplinary disease management programs. Device-based therapies, such as implantable cardioverter-defibrillators (ICD) or cardiac resynchronization therapy (CRT-D/CRT-P), are recommended for secondary prevention of ventricular arrhythmias or primary prevention in patients with LVEF < 35% despite optimized therapy [1].

Within the broad spectrum of HF phenotypes, treatment efficacy can be improved by tailoring therapy to individual patient characteristics, including gender, comorbidities, treatment response, side effect tendencies, physical activity tolerance, and frailty status [9,10]. Within this context, precision medicine is conceptualized as a personalized approach to the management of diseases that takes the complex interplay between a patient’s genetic makeup, environmental influences, and lifestyle habits into consideration.

### 1.3. The Need for Personalization in Heart Failure Therapy

The notion of a “one-size-fits-all” approach has certainly had its benefits for many, but growing evidence supports a more personalized medicine approach to optimize outcomes and minimize adverse effects. Indeed, genetic variants and epigenetic modifications have been shown to influence both drug response and patient prognosis. Key tools include the omics sciences (genomics, transcriptomics, proteomics, metabolomics, and epigenomics), as well as pharmacogenomics (the branch of genetics that examines how genetic variants influence drug efficacy and safety), and artificial intelligence and machine learning (the study of computer-based learning systems). These technologies have the potential to facilitate predictive diagnostics and individualized therapy selection [11]. Metabolomics, which investigates small molecules from metabolic pathways, has been proposed as a potential source of novel biomarkers and a tool for HF phenotyping, with the capacity to enhance diagnosis, prognosis, and treatment monitoring [9,12].

### 1.4. Overview of Genetic Influence in Disease Progression and Treatment Response

The pivotal role of familial predisposition and the importance of longitudinal follow-up were first emphasized by the Framingham Study, which constituted the basis for contemporary cardiovascular epidemiology. Indeed, the development of multigenerational cohorts, in conjunction with the systematic monitoring of genetic and environmental determinants, has been instrumental in highlighting the potential influence of genetic susceptibility on the development and progression of heart failure [13].

In recent years, the landscape of phenotype- and genotype-specific therapies has evolved rapidly. Indeed, variations in genes involved in pharmacokinetic and pharmacodynamic mechanisms are the focus of numerous studies exploring their association with the therapeutic response to specific drugs [14].

Moreover, the “second hit” theory suggests that genetic predispositions (e.g., *TTN* variants) interact with external factors to trigger cardiomyopathies, including alcohol-induced, chemotherapy-related, or peripartum cardiomyopathy (PPCM). Truncating variants in *DSP*, *FLNC*, and *BAG3* have also been identified in patients with PPCM [15,16,17].

In inherited cardiomyopathies, specific gene mutations (e.g., *LMNA*, *DSP*, *DSG2*, *RBM20*, *FLNC*, *PLN*) are associated with an increased risk of life-threatening arrhythmias, supporting early ICD implantation in some phenotypes [18]. 

Epigenetic modifications, triggered by environmental or stress-induced factors (e.g., DNA methylation, histone modification), are increasingly recognized as contributors to HF pathophysiology. These alterations may also serve as early, non-invasive biomarkers for disease diagnosis, progression, and prognosis [19]. 

Additionally, studies have demonstrated the involvement of microRNAs (miRNAs) in the pathophysiology of HF, and the regulation of these molecules has been shown to have promising therapeutic effects [20]. Future therapeutic strategies may include gene silencing, replacement, or correction; however, further research is needed to evaluate their safety, clinical applicability, and ethical implications. 

## 2. Genetic Mechanisms and Therapeutic Response in Heart Failure

### 2.1. Key Genetic Factors: Single-Nucleotide Polymorphisms, Copy Number Variations, and Mutations

The genetic architecture that influences the risk, progression, and therapeutic response in HF is complex and involves both common variants and rare mutations [21]. 

SNPs are the most common type of genetic variation [22]. While many SNPs have small individual effects, some are associated with clinically relevant phenotypes [22]. For instance, SNPs in genes that encode neurohormonal receptors, ion channels, or cytokines can modify disease severity and influence how patients respond to therapeutic interventions [23]. 

Copy number variations (CNVs) have received comparatively less attention in the context of HF. However, CNVs can induce specific cardiomyopathies (for instance, large gene deletions in *dystrophin* result in Duchenne cardiomyopathy, associated with HF) [24,25].

In general, common variants are expected to display an additive effect with respect to the phenotypic outcome, which depends on the overall genetic profile rather than from the effect of an individual variant.

In contrast to common variants, rare, high-impact variants in cardiac genes have been shown to be pathogenic and closely associated with familial cardiomyopathies that may progress to HF. One notable example is truncating mutations in the titin *(TTN)* gene, which encodes a giant sarcomeric protein. *TTN* truncating mutations have been detected in 20–25% of dilated cardiomyopathy (DCM) cases, thereby constituting a risk factor for the development of systolic HF [25]. 

Another key gene is *LMNA*, which encodes nuclear lamins. Missense or truncating mutations in *LMNA* are associated with an aggressive form of DCM and a predisposition to the development of malignant ventricular arrhythmias and sudden cardiac death [26,27]. 

Numerous other genes have been implicated in inherited cardiomyopathies and the development of HF. Examples include *MYH7* (beta-myosin heavy chain) and *MYBPC3* in hypertrophic cardiomyopathy; *RBM20* and *FLNC* in dilated cardiomyopathy; and *DSP/PKP2* in arrhythmogenic cardiomyopathy. Each of these genes confers a variable degree of risk of HF or arrhythmia [28]. 

Recent large-scale studies have provided a more integrated view of HF genetics. A 2025 genome-wide meta-analysis involving over 2.3 million individuals identified 176 risk loci for HF, organized into functional modules related to obesity, blood pressure, atherosclerosis, immune regulation, and arrhythmias. Furthermore, rare variants in cardiomyopathy genes, including *TTN*, *MYBPC3*, *BAG3*, and *FLNC*, have been identified as significant contributors to high HF risk. It is notable that polygenic risk scores have been demonstrated to modify the penetrance of monogenic mutations, suggesting an interplay between rare and common variants. The present findings underscore the implications of both rare and polygenic mechanisms in HF, thus emphasizing the potential clinical utility of combining rare variant screening with polygenic risk stratification [21,28].

Advancements in sequencing technologies have led to significant progress in our capacity to map this complex landscape [29].

In this context, next-generation sequencing (NGS) represents a pivotal instrument in the evaluation of inherited cardiomyopathies. It is notable that techniques such as whole-exome sequencing (WES) and whole-genome sequencing (WGS) allow for the simultaneous detection of pathogenic variants, variants of uncertain significance (VUS), and structural alterations such as copy number variations (CNVs). Nevertheless, the interpretation of variants remains a significant challenge. In order to ensure a standardized assessment, variants are classified according to the ACMG/AMP guidelines into five categories (from benign to pathogenic). A multifactorial approach is employed, integrating population frequency data (e.g., gnomAD), in silico predictions, functional evidence, and clinical correlations. In highly variable genes like *TTN*, where truncating variants may be present in asymptomatic individuals, interpretation must account for exon usage and transcript expression levels to avoid overestimating pathogenicity. Consequently, precise interpretation necessitates the integration of genetic findings with clinical context, a process that is essential to support genetic counselling, risk stratification, and therapeutic decision-making in HF [30].

### 2.2. Epigenetic Modifications and Their Impact on Heart Failure

In addition to DNA sequence variations, epigenetic modifications have been demonstrated to play a role in the pathogenesis of HF and in drug response. 

The focus of epigenetics research is on reversible chemical alterations to DNA and histones that regulate gene expression. Key epigenetic mechanisms include DNA methylation, post-translational histone modifications (acetylation, methylation, phosphorylation, ubiquitination), chromatin remodeling, and non-coding RNA-mediated regulation [31,32,33]. 

There is increasing evidence that, in the case of HF, pathological stressors such as hemodynamic overload, neurohormonal activation, ischemia, or inflammation induce epigenetic changes that alter cardiomyocyte gene expression profiles [31,32]. 

It is important to emphasize that epigenetic marks have the potential to be reversed, thus rendering them suitable therapeutic targets. 

Histones are small, conserved proteins that organize DNA into nucleosomes, with core histones (H2A, H2B, H3, H4) forming an octamer around which DNA wraps. Histone H1 stabilizes the structure by linking nucleosomes. Post-translational modifications of histone tails, such as acetylation and methylation, regulate chromatin structure and gene expression [34,35]. 

In heart injury, tissue-resident fibroblasts are the main contributors to cardiac fibrosis. They first proliferate, then activate genes for contraction and produce extracellular matrix proteins. Histone modifications have been shown to regulate this fibrotic process through epigenetic mechanisms [36]. In a mouse model of HF, HDAC1 (Histone Deacetylase 1) levels were found to be elevated in cardiac fibroblasts [37]. 

Treatment with a class I HDAC inhibitor reduced fibrosis and improved heart function by blocking fibroblast proliferation and activation, and by inducing apoptosis. In vitro, these inhibitors increased cell cycle inhibitors (p21, p17) and the proapoptotic protein p53. They also suppressed key profibrotic factors like CTGF, ECM proteins, and IL-6 [38].

However, the specific effect on histone acetylation was not analyzed, leaving open the possibility of non-histone targets. Additionally, BET (Bromodomain and Extra-Terminal domain proteins) protein inhibitors like JQ1 have shown beneficial effects in various HF models [39]. 

While histone acetylation has been studied in cardiac fibrosis, the role of histone methylation remains largely unexplored and warrants further research [32]. 

Changes in chromatin conformation can influence gene expression by modifying how frequently genes interact with distant regulatory elements. This regulation is important in many biological processes, including heart function. Two recent studies investigated this in the heart using Hi-C, a technique that maps 3D genome organization. They analyzed cardiomyocytes from mice lacking the CTCF protein, a key regulator of chromatin structure, after inducing pressure overload through transverse aortic constriction. The results showed that disrupting chromatin architecture was associated with significant changes in gene expression in response to cardiac stress [40,41,42].

Recent studies highlight the role of chromatin architecture in HF, particularly through mutations in the *LMNA* gene, which encodes lamin A/C, a key structural protein of the nuclear envelope [43]. A frameshift mutation (K117fs) led to reduced lamin A/C levels, disruptions in lamina-associated domains (LADs), and changes in histone methylation patterns (e.g., H3K9me2 and H3K4me3), resulting in increased expression of genes in the PDGF pathway, linked to arrhythmias. Another mutation (K219T) caused electrical conduction defects by increasing H3K27me3 on the *SCN5A* gene promoter, reducing its expression, and relocating it to the nuclear periphery. These findings support the idea that the nuclear lamina helps organize chromatin to regulate proper gene expression in heart cells [44]. However, the exact mechanisms by which chromatin structure shapes epigenetic marks remain unclear [32]. 

A key recent discovery in HF research is the interaction between epigenetic enzymes and long non-coding RNAs (lncRNAs). One lncRNA, Mhrt, was found to protect the heart from hypertrophy by binding to the chromatin remodeler BRG1, preventing it from attaching to DNA and activating stress-related genes. In mouse models subjected to pressure overload, overexpression of Mhrt improved heart function by blocking the pathological switch from *MYH6* to *MYH7*, a marker of hypertrophy [32]. 

These findings support the potential for epigenetics-based therapies in HF. Inhibitors of class I and II HDACs have been shown to reduce cardiomyocyte hypertrophy and fibrosis, improving heart function in mouse models [45]. Additionally, BET inhibitors such as JQ1 are being explored as promising treatments for HF [46]. 

Current epigenetic drugs have limitations for treating HF due to their pleiotropic effects, as their targets are widely expressed in many tissues. Moreover, the same epigenetic enzymes involved in HF are also linked to cancer and neurological diseases. To make epigenetic therapies more effective and specific for HF, it is crucial to identify HF-specific epigenetic networks. This could lead to the development of targeted treatments, such as combinations of selective enzyme inhibitors or multi-target drugs that specifically act on epigenetic enzymes involved in HF [32]. 

Gene therapy can also be viewed from the perspective of epigenetics. CRISPR-based epigenome editing involves fusing a deactivated Cas9 to epigenetic modifiers to write or erase epigenetic marks at specific genes. This technique has been proposed as a means of activating silenced genes or repressing overactive genes without causing permanent alterations to the genome [47]. 

In conclusion, although there is considerable potential for CRISPR-based epigenetic editing, further research is essential to fully exploit its therapeutic capabilities and make these innovations accessible and safe in the context of cardiovascular diseases [48,49].

**Figure 1:**
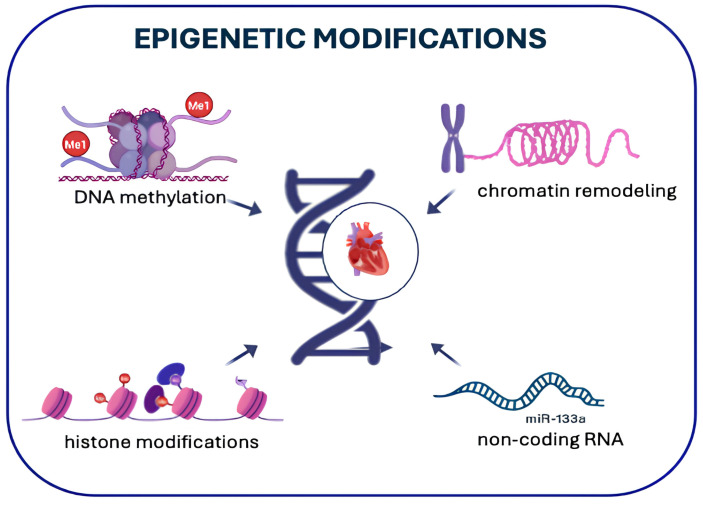
Epigenetic Modifications. The figure summarizes the main mechanisms involved in epigenetic modification, including DNA methylation, histone modifications, chromatin remodeling, and non-coding RNA regulation, which collectively influence gene expression without altering the DNA sequence.

Figure 1 describes the main mechanisms involved in epigenetic modification.

### 2.3. Genetic Variability: A Key Determinant of Treatment Efficacy

Individual genetic variability has been identified as a key factor influencing the efficacy of HF therapies. Research in this area has shown that patients respond in a variety of ways to standard therapy, even when administered under consistent conditions of dosing and adherence [50,51,52]. This variability in treatment response is frequently attributed to genetic polymorphisms that alter drug pharmacodynamics or pharmacokinetics. For instance, common SNPs in the β_1_-adrenergic receptor gene (*ADRB1*) have been shown to modify patient responses to β-blockers [53]. Similarly, polymorphisms in the angiotensin-converting enzyme (ACE) gene and the angiotensinogen gene have been associated with differential responses to ACEi or angiotensin receptor blockers, although findings have been heterogeneous [54].

A recent study found that a polymorphism in the sodium–glucose cotransporter 2 gene (*SLC5A2*) was associated with variable clinical outcomes in patients treated with the SGLT2 inhibitor dapagliflozin, suggesting that gene variants can influence the therapeutic benefits of this class of drugs [55].

In addition, variations in genes involved in drug metabolism and transport can lead to altered plasma drug concentrations, impacting both efficacy and toxicity. 

For instance, the primary metabolism of β-blockers such as metoprolol is primarily mediated by the cytochrome P450 (CYP) 2D6 enzyme. Individuals with CYP2D6 poor metabolizer alleles exhibit higher plasma drug levels for a given dose compared to ultra-rapid metabolizers, potentially resulting in exaggerated β-blockade or adverse effects [14].

Recognizing these influences, there is growing interest in pharmacogenomic profiling within cardiovascular medicine. Identifying DNA variants that affect drug response may enable clinicians to predict individual benefit, anticipate adverse reactions, and optimize dosing strategies, thereby contributing to more precise and effective HF management [29].

### 2.4. Gene-Environment Interactions and Their Impact on Drug Metabolism

The clinical outcomes of HF are also determined by a complex interplay between genetic and environmental factors, impacting various physiological processes, including metabolism and drug response. 

For instance, CYP3A4, an enzyme involved in the metabolism of cardiovascular drugs, exhibits significant interindividual variability [56].

In the event of a patient with a genetic variant that reduces CYP3A4 activity being prescribed drugs such as ivabradine or amlodipine, it is important to note that their tolerance of these medications may be subject to variation depending on other factors such as grapefruit juice consumption or the concomitant use of medications that interact with CYP3A4 [56]. These interactions have the capacity to induce alterations in drug levels within the body, increasing the risk of therapeutic failure or toxicity. 

Another relevant aspect of gene–environment interplay pertains to organ function and disease state. The co-occurrence of HF and renal or hepatic impairment (environmental factors in a broad sense) is well documented, with the potential to modify drug handling regardless of genotype [57,58].

HF pharmacogenomics considers not only drug–gene interactions, but also lifestyle factors and exposure to harmful substances, which can modulate drug efficacy. For instance, smoking induces the CYP1A2 enzyme, potentially altering the metabolism of drugs in HF patients who smoke [59].

The *GRK5* Leu41 variant is a well-described example of gene-environment interplay. This variant enhances β-adrenergic receptor desensitization in response to catecholamines, potentially diminishing the need for β-blockers in HF [60].

## 3. Pharmacogenomics in Heart Failure: The Role of Genetic Variations in Drug Response

### 3.1. Overview of Pharmacogenomics in Heart Failure

Pharmacogenomics, which investigates the influence of genetic variation on drug efficacy and safety, has become increasingly interesting in the management of HF. Personalizing pharmacological treatments based on an individual’s genetic profile offers the potential to improve therapeutic outcomes and reduce the incidence of adverse drug reactions [14,61,62,63,64].

Although preliminary findings are encouraging, the field of pharmacogenomics still faces significant challenges. These include the limited reproducibility of genetic associations across different populations, along with a lack of randomized controlled trials and cost-effectiveness analyses, and this constrains the clinical translation and broader implementation of genotype-guided strategies in HF management [61,62].

The following sections present a review of key pharmacogenomic evidence for common classes of HF medications, along with a discussion of future directions.

### 3.2. Impact on Common HF Medications

#### 3.2.1. ACE Inhibitors (ACEi) and Angiotensin Receptor Blockers (ARBs)

For ACEi and ARBs, interindividual variability in response has been attributed, at least in part, to genetic variants within the RAAS. The insertion/deletion (I/D) polymorphism of the *angiotensin-converting enzyme (ACE) gene* is one of the most studied variants [61]. The deletion allele (D) has been associated with higher levels of circulating ACE and has been linked to worse HF outcomes at baseline [65,66]. Furthermore, some studies suggest that the *ACE I/D* genotype may modulate the response to ACEi, for example, by influencing the degree of left ventricular remodeling [67,68]. However, results have been controversial and inconsistent [69,70,71]. 

This finding indicates that, while the *ACE D* allele may potentially contribute to a more active renin–angiotensin system, the impact of this variant could be influenced by polygenic influences, which complicates the interpretation of results.

An early study investigated the potential impact of a specific genetic variation in the *AGTR1* gene (A1166C) on the response of patients with HF to candesartan. This finding suggested the potential involvement of genetics in determining treatment response [72].

Nevertheless, this potential association remains unconfirmed by larger studies. 

A more extensive pharmacogenomic investigation, encompassing over 2700 patients, as part of the CHARM trial, failed to identify any substantial genetic indicators associated with candesartan’s efficacy or safety [73]. This prompts further consideration of previous findings and underscores the complexity of genetic influences on drug response in HF.

Moreover, pharmacogenomics has facilitated a more comprehensive understanding of the adverse effects associated with ACEi. Polymorphisms in the *bradykinin B2 receptor (BDKRB2) gene* have been shown to be consistently associated with ACE inhibitor-related cough [74]. Other variants are also implicated, for example, the *carboxypeptidase N subunit 2 (CPN2) gene*, which plays a regulatory role in kinins [74]. 

Genetic susceptibility has been demonstrated to be associated with ACEi-induced angioedema. Research has identified common genetic variants in the proximity of the *bradykinin B2 receptor (BDKRB2) gene*, which have been linked to an elevated risk of this adverse reaction [12,71,72,73,74,75,76].

In summary, pharmacogenomics research has identified biologically plausible variants that contribute to response variability to ACEi and ARBs. However, further evidence is required from larger, multi-ethnic studies and clinical trials.

#### 3.2.2. Angiotensin Receptor-Neprilysin Inhibitor (ARNI)

Some data suggest that genetic variability can significantly influence individual responses to sacubitril/valsartan therapy. The *CES1* gene, which is responsible for encoding carboxylesterase 1, is critical for the hepatic activation of sacubitril. The G143E (rs71647871) loss-of-function variant has been demonstrated to significantly reduce *CES1* activity, thereby potentially diminishing drug activation and therapeutic benefit [76]. 

Another small trial showed that genetic polymorphisms in the gene encoding neprilysin, the pharmacological target of sacubitrilate, have the potential to alter enzyme expression or function. Specifically, the rs701109 variant has been linked to a reduction in the efficacy of treatment with ARNI [77]. 

The finding was in line with previous research indicating that variations in the neprilysin gene can influence both the effectiveness of therapeutic interventions and the long-term safety of patients [78]. 

In addition, variants in hepatic uptake transporters, including SLCO1B1 and SLCO1B3, have been demonstrated to be associated with altered valsartan pharmacokinetics, with the potential to influence overall ARNI exposure; however, given the absence of evidence from cell lines and preclinical models, further research is necessary to substantiate these findings [79]. 

Collectively, these studies support the potential role of pharmacogenetics in optimizing sacubitril/valsartan therapy and emphasize the necessity for further research to guide precision medicine in HF management.

#### 3.2.3. β-Blockers

Although beta-blockers improve outcomes in patients with HF, individual responses are variable [1,80,81]. Since clinical factors cannot fully explain this variability, attention has shifted to pharmacogenetics [82]. Research has focused on genetic variants of pharmacodynamic genes, particularly *ADRB1*, *ADRB2*, *ADRA2C*, *GRK4*, and *GRK5*, which modulate beta-adrenergic signaling and receptor desensitization [83,84,85,86,87,88].

However, the results were considered inconsistent and insufficient for clinical recommendations, in contrast to the well-established role of CYP2D6 in metoprolol pharmacokinetics [14].

Some evidence supports the hypothesis that *GRK4* variants may function as endogenous beta-blockers by enhancing receptor desensitization [14,83].

Other variants, such as *ADRB1* Arg389Gly, the Ser49-Arg389 haplotype, and *ADRA2C* Del322-325, showed trends consistent with reduced benefit of beta-blockers in carriers of alleles associated with reduced adrenergic function; however, correction for multiple testing showed no significance [14,86].

For example, patients homozygous for the Ser49-Arg389 haplotype with increased adrenergic function appeared to derive the greatest survival benefit, and those with *ADRA2C* Del322-325 homozygosity also showed a favorable response, especially among African Americans [14,89].

Furthermore, GWAS have indicated that the most significant genetic predictors of beta-blocker response lie outside of traditional candidate genes, highlighting the limitations of the candidate gene approach [90,91].

In summary, larger and more diverse studies are needed to confirm and extend these findings [14,89].

#### 3.2.4. Mineralocorticoid Receptor Antagonists

In recent years, several studies have explored how genetic variants influence the response to MRAs drugs, such as spironolactone, eplerenone, and finerenone, in patients with HF or chronic kidney disease.

A 2020 study on Egyptian patients with HF reported that *AGT* rs699 and *CYP11B2* rs1799998 polymorphisms, in combination with baseline potassium, significantly affected the clinical response to spironolactone, explaining a substantial portion of the variability in improvements in ejection fraction and potassium levels. However, these findings require replication in larger and more diverse populations [92].

A 2021 study analyzed the association between the rs5522 polymorphism of the *NR3C2* gene and the response to spironolactone in Aldo-DHF Trial participants with diastolic HF.

The study found that spironolactone can attenuate the progression of diastolic dysfunction more effectively in individuals associated with the G allele of rs5522. This suggests that the rs5522 variant could serve as a prognostic marker to identify patients with HF who are more likely to benefit from spironolactone. However, further research is needed to confirm these results and determine if they extend to different populations [93].

A 2022 study provided novel insight into the mechanism of action of finerenone. The study demonstrated that the kinase GRK5 is essential for the inhibition of the cardiac mineralocorticoid receptor by finerenone, but not by eplerenone. Finerenone stimulates GRK5 phosphorylation of the receptor, suppressing its transcriptional activity and thereby enhancing cardio protection [94].

These findings suggest that genetic variations or reduced expression of GRK5 may influence the therapeutic efficacy of finerenone.

Collectively, these studies highlight the clinical relevance of genetic variants in modulating the response to MRAs, supporting the potential for personalized therapeutic strategies in HF management.

#### 3.2.5. SGLT2 Inhibitors

Emerging evidence also suggests that genetic variants may influence responses to SGLT2i. In a 2021 study, Katzmann et al. demonstrated that variants in the *SLC5A2* gene, associated with reduced SGLT2 expression, were correlated with a lower risk of HF. The protective effect is independent of diabetes and is mediated by metabolic improvements. These findings support the hypothesis that SGLT2i may have a direct cardioprotective effect, preventing HF [95].

This suggests that, in the future, genetic profiling may help identify patients most likely to benefit from SGLT2i therapy, facilitating greater personalization of treatment.

In a 2025 clinical study, Abou Warda et al. investigated the rs3813008 polymorphism of the *SLC5A2* gene in patients with HF. They found that this variant was associated with a lower risk of cardiovascular events in patients not treated with SGLT2i, but a higher risk in those treated with dapagliflozin. This suggests a possible genotype-drug interaction that warrants further studies to substantiate these findings, and better analyze the potential for pharmacogenomic testing to personalize SGLT2i therapy in HF management [55].

From a pharmacokinetic perspective, polymorphisms in genes involved in metabolizing SGLT2i appear to influence its plasma levels. For instance, the *UGT1A93* and *UGT2B42* alleles have been observed to result in increased exposure to canagliflozin, although with no clear clinical implications for safety [96]. Conversely, dapagliflozin is predominantly metabolized by UGT1A9 and UGT2B4 (with a minor contribution from CYP3A4) [97,98], suggesting that variants in these enzymes may modify its pharmacokinetics and, consequently, the therapeutic response.

### 3.3. Future Directions: Integrating Pharmacogenomic Data into Clinical Practice

While many genetic variants have been linked to differences in drug response in HF, strong evidence from prospective studies is still needed to confirm these associations. Much of the current data comes from retrospective analyses or specific patient subgroups.

Future studies should also explore the broader genetic landscape, including polygenic risk scores, genetic biomarkers, and gene–gene interactions [99]. 

The successful incorporation of pharmacogenomics into the management of HF will necessitate collaborative approaches, multimodal assessments incorporating AI-enabled diagnostics and genetic testing, patient engagement, and the responsible use of genetic information.

## 4. Gene Therapy in Heart Failure: Emerging Strategies for Targeted Treatment

### 4.1. The Rationale for Gene Therapy in Heart Failure

Although modern pharmacological treatments and medical devices have improved HF management, the condition is still associated with a high rate of morbidity and mortality due to its complex underlying pathophysiology. In this challenging context, gene therapy is emerging as a potentially transformative approach, thanks to its ability to target and modify faulty genes at the molecular level [49]. Approaches such as antisense oligonucleotides (ASOs), small interfering RNAs (siRNAs), and CRISPR-Cas9 have shown encouraging results in regulating gene expression, typically with limited side effects [100,101].

Among the available vectors, adeno-associated viruses (AAVs) have become the gold standard for in vivo gene delivery, largely due to their strong affinity for cardiac tissue, sustained expression, and low immunogenicity [102]. These technologies are opening promising new avenues for treating both genetic and non-genetic forms of HF [103,104].

### 4.2. Gene Therapy Approaches in Heart Failure

Gene therapy strategies for HF are generally classified into three main strategies: gene silencing, gene replacement, and gene editing. Each operates via distinct mechanisms, targets specific molecular pathways, and is progressing at a different pace in clinical development. Among these, gene silencing, particularly through ASOs and siRNAs, has shown the most clinical progress. These molecules work by binding selectively to messenger RNA (mRNA), thereby blocking the production of proteins linked to disease [49,105].

Some siRNA-based treatments have already entered clinical use. One of the most significant examples is patisiran, the first siRNA therapy approved by both the FDA and EMA for treating hereditary transthyretin amyloidosis (ATTRv), which has also demonstrated positive effects on cardiac symptoms [106,107].

Similarly, vutrisiran has shown efficacy in phase 3 trials, offering benefits for both the neurological and cardiac manifestations of ATTR [108,109].

These therapies are delivered specifically to hepatocytes, where transthyretin is primarily produced, using delivery platforms like lipid nanoparticles or N-acetylgalactosamine (GalNAc) conjugates. This delivery strategy has become a clinically validated example of gene silencing for systemic contributors to HF. In parallel, ASOs such as inotersen have entered clinical practice as well, showing effectiveness in alleviating neuropathic symptoms in ATTRv and providing some benefit in cardiac involvement [110]. However, the use of ASOs is somewhat limited by safety concerns, including risks like thrombocytopenia and glomerulonephritis, which require careful monitoring of patients [49].

In contrast, gene silencing strategies that act directly on the heart, such as anti-microRNA (anti-miR) therapies, are still in early clinical development. One such example is CDR132L, an anti-miR-132 compound aimed at reducing cardiac hypertrophy and fibrosis. It has demonstrated a good safety profile and preliminary efficacy in a phase 1b trial in patients with chronic HF [111], although it remains investigational. Similarly, microRNA mimics like miR-199a-3p and miR-590-3p, which aim to stimulate cardiomyocyte proliferation, have shown promise in animal models but have yet to move into human trials [112,113].

Gene replacement strategies primarily focus on inherited cardiomyopathies caused by loss-of-function mutations. These approaches often rely on AAV vectors and are still in experimental stages. Several candidates are being explored in clinical trials, including AAV-mediated delivery of *MYBPC3* for hypertrophic cardiomyopathy (HCM) and *LAMP2B* for Danon disease [114,115].

In addition to AAVs, other viral vectors have been explored for cardiac gene therapy, although with more limited success. Adenoviral vectors (primarily Ad5) were among the first utilized due to their high transduction efficiency and large transgene capacity. However, their clinical applicability has been limited by strong immunogenicity and transient gene expression. Lentiviral vectors have been shown to facilitate stable genomic integration; however, they have also been associated with a high risk of insertional mutagenesis and insufficient cardiac tropism [49].

In recent years, significant innovations in synthetic AAV capsids have emerged. 

Engineered capsids, including AAVMYO, AAV.KK04, and Anc80, have demonstrated superior cardiac transduction efficiency and reduced immunogenicity in comparison to natural serotypes. Specifically, AAV.KK04 has shown high myocardial selectivity and effective liver detargeting in both murine and non-human primate models. [116].

The combination of these advances with cardiac-specific promoters and immune evasion strategies is resulting in an expansion of the potential of viral delivery platforms for the treatment of HF.

Though none have received regulatory approval yet, they represent advanced preclinical or early-stage efforts using cardiac-tropic AAV serotypes and promoters tailored for specific heart cell types [117].

Another exciting area is modified mRNA (modRNA) technology, designed for short-term protein expression to promote tissue repair and protect the heart. In preclinical studies, delivering modRNA encoding proteins such as VEGF-A, IGF-1, and follistatin-like 1 has led to improved cardiac function after myocardial infarction [118,119].

One such therapy, AZD8601 (modRNA for VEGF-A), has advanced to phase 2 clinical trials in patients undergoing coronary artery bypass grafting (CABG) and has been shown to be safe and well tolerated [120].

Still, most modRNA therapies are in early stages. A particularly novel direction is using modRNA to generate transient CAR-T cells in vivo that can selectively target activated fibroblasts, with the goal of reducing cardiac fibrosis. This strategy has demonstrated efficacy in mouse models of HF but is still experimental [121].

Gene editing techniques, especially CRISPR-Cas9, hold great potential for correcting mutations or disrupting harmful pathways with high precision. Although promising, these tools are largely still in preclinical stages. In animal studies, CRISPR-Cas9 has been applied to inherited cardiomyopathies and to inactivate genes like CaMKIIδ, which contribute to maladaptive remodeling [122].

Newer platforms such as base editing and prime editing aim to refine this approach further by avoiding double-stranded DNA breaks, improving safety and precision [123,124].

A notable clinical exception is NTLA-2001, a CRISPR therapy targeting the *TTR* gene, which has progressed into clinical trials. Initial results suggest it can reduce serum TTR levels effectively and is generally well tolerated [125].

Despite these advances, cardiac-targeted gene therapies, whether for silencing, replacement, or editing, remain largely experimental. In contrast, liver-directed strategies addressing systemic HF contributors like TTR and PCSK9 are already in clinical use. Moving cardiac gene therapies into broader clinical application will require continued innovation, careful safety evaluations, and long-term studies [49].

Table 2 summarizes the main gene therapy strategies currently in development or that have been approved for the treatment of HF.

### 4.3. Challenges in Translating Gene Therapy to Clinical Practice: Safety, Efficacy, and Ethical Considerations

Despite notable advances, several critical barriers continue to hinder the clinical translation of gene therapy in HF. Among the most pressing safety concerns are immunogenicity, off-target effects, and the potential for insertional mutagenesis, particularly when using viral vectors or genome-editing tools like CRISPR-Cas9 [122,124,134].

Another major challenge involves the efficiency of gene delivery, especially to cardiac tissue. This is further complicated by difficulties in achieving targeted delivery and sustaining long-term gene expression [134,135].

Beyond the scientific and technical obstacles, the path to clinical adoption is also shaped by regulatory and logistical complexities, including the complexity of vector design, limitations in large-scale manufacturing, and the high cost of these therapies. At the same time, ethical considerations, such as concerns over germline editing and inequitable access, call for clear regulatory frameworks and open, patient-centered communication [131,136].

## 5. Specific Heart Failure Phenotypes

### 5.1. Genetic Basis and Therapeutic Implications in Dilated Cardiomyopathy

DCM is a common myocardial disorder, affecting approximately 1 in 250 individuals in the general population [18,25,137]. It represents a final common phenotype resulting from a wide array of genetic and acquired factors and is characterized by left ventricular dilation and systolic dysfunction in the absence of abnormal loading conditions or coronary artery disease [26,138]. Clinically, DCM encompasses a spectrum of clinical presentations, including familial and sporadic forms, as well as intermediate phenotypes such as hypokinetic non-dilated cardiomyopathy and isolated left ventricular dysfunction [137,139,140]. 

As stated, approximately 30–50% of DCM cases are familial, with autosomal dominant inheritance being the most common, though autosomal recessive, X-linked, and mitochondrial patterns are also described [140]. Genetic testing identifies pathogenic or likely pathogenic variants in 30–40% of familial DCM and 15–25% of sporadic cases [25,138]. 

Beyond single-gene mutations, polygenic inheritance and gene-environment interactions are increasingly recognized as key determinants of disease expression and penetrance. Notably, up to 15% of patients with acquired DCM (e.g., alcohol-related, peripartum, or myocarditis-associated) carry rare pathogenic variants, particularly in *TTN*, suggesting that a genetic predisposition may sensitize the myocardium to external stressors [25].

From a phenotype point of view, the therapeutic management of DCM with reduced LVEF has traditionally relied on standardized pharmacological approaches aimed at modulating neurohormonal dysregulation. However, advances in cardiovascular genetics and molecular imaging have shown that the clinical course, arrhythmia risk, and treatment response in DCM vary significantly depending on the underlying genotype [18,141].

Among sarcomeric genes, truncating variants in *TTN (TTNtv)*, which encodes titin, a giant protein contributing to sarcomeric elasticity, are the most frequently implicated, accounting for up to 20% of all DCM and more than 25% in familial forms [25].

These variants have been associated with a high prevalence of left ventricular reverse remodeling under optimized GDMT [25,141], and these patients often experience substantial improvement in systolic function and symptom burden, particularly when treatment is initiated at early disease stages [141].

Mutations in *LMNA*, encoding nuclear envelope protein lamin A/C, are found in 5-10% of familial DCM and are associated with a malignant arrhythmic phenotype. These patients typically present at a younger age with conduction system disease (e.g., atrioventricular block, left bundle branch block), non-sustained or sustained ventricular tachycardia, and rapidly progressive LV dysfunction [26]. Importantly, *LMNA* mutations are associated with a high risk of SCD, often independent of the degree of LV impairment, supporting early ICD implantation even in patients with preserved LVEF [27].

In this context, the use of genotype-specific risk models, such as the LMNA Risk-VTA score, allows clinicians to identify patients who may benefit from primary prevention ICD implantation despite an LVEF above the conventional threshold [141].

Cytoskeletal and desmosomal gene mutations further expand the phenotypic spectrum. Truncating mutations in *FLNC (filamin-C),* a cytoskeletal scaffolding protein, are increasingly recognized and associated with high arrhythmic risk, frequent myocardial fibrosis, and a relatively poor prognosis, even in the presence of modest ventricular dysfunction [142], supporting early device implantation irrespective of LVEF, particularly when late gadolinium enhancement (LGE) is present on CMR imaging [137,142].

Similarly, *DSP (desmoplakin)* mutations, typically associated with arrhythmogenic right ventricular cardiomyopathy (ARVC), may present with left-dominant arrhythmogenic DCM, often showing extensive subepicardial fibrosis, frequent premature ventricular contractions, and frequent progression to HF with preserved or mildly reduced LVEF [139]. In such cases, LGE on CMR often reveals a distinctive non-ischemic pattern that precedes systolic dysfunction and strongly correlates with arrhythmic risk [142]. Inflammatory changes may be present, and clinical overlap with myocarditis can complicate diagnosis, sometimes leading to inappropriate administration of immunosuppressive therapy [18].

The *RBM20* gene, encoding an RNA-binding protein involved in splicing of sarcomeric transcripts including TTN, is associated with early-onset, aggressive DCM, often with high arrhythmogenic burden. Affected patients frequently present in their twenties or thirties with both systolic dysfunction and malignant ventricular arrhythmias, necessitating early electrophysiological monitoring [140], with a low threshold for ICD implantation [142].

Variants in ion channel-related genes (*SCN5A*, *HCN4*) can also manifest with DCM, often overlapping with conduction defects, sinus node dysfunction, and atrial arrhythmias.

Looking ahead, the therapeutic landscape of genetic DCM is poised to expand beyond symptomatic management toward molecular targeting of pathogenic mechanisms [18,142]. Gene therapy approaches, ASOs, RNA interference, and CRISPR-based gene editing, are under development for mutations in *TTN*, *LMNA*, and *RBM20* [142]. In particular, adeno-associated virus (AAV)-mediated gene transfer and adenine base editing have shown promise in preclinical models for *RBM20*-related DCM [143,144]. These technologies aim to correct or suppress pathogenic transcripts, restore normal protein function, or compensate for loss-of-function alleles [142]. For *LMNA* mutations, antisense oligonucleotides (ASOs) and CRISPR-Cas9-based strategies are being explored to silence or edit dominant-negative alleles [145]. Although still in early-phase trials, preliminary data are encouraging and suggest that targeted molecular interventions may eventually transform the management of selected genetic subtypes [18,142]. In parallel, pharmacologic strategies are being developed to reduce myocardial fibrosis (e.g., NLRP3 inflammasome inhibitors), stabilize sarcomeric proteins (e.g., omecamtiv mecarbil, mavacamten), or modulate calcium homeostasis (e.g., L-type calcium channel blockers in arrhythmogenic forms of DCM) [146,147,148], with potential application in arrhythmogenic forms of DCM [10].

In summary, the therapeutic implications of DCM are intimately linked to its genetic substrate [18,137,141]. While GDMT remains the foundation of care, integrating genetic and imaging biomarkers into clinical decision-making enables a precision approach that aligns therapy with individual risk profiles [18,141]. The identification of high-risk genotypes informs the early use of device therapy, guides family screening, and facilitates the enrollment of patients into emerging trials of gene-targeted therapies [18,137,141].

Table 3 summarizes the major correlations between pathogenic genetic variants and clinical phenotypes in DCM. It highlights the response to GDMT, the associated arrhythmic risk and the related therapeutic implications.

### 5.2. Hypertrophic Cardiomyopathy (HCM)

HCM is defined as a primary myocardial disorder characterized by unexplained left ventricular hypertrophy in the absence of secondary causes. It is now recognized as the most common inherited cardiovascular disorder, affecting 1 in 200 to 1 in 500 of the general population [149]. It is estimated that 30-40% of HCM cases have a genetic basis, usually characterized by autosomal dominant transmission with incomplete penetrance and variable expressivity. Most pathogenic variants are in eight sarcomeric genes, including *MYH7*, *MYBPC3*, *TNNT2*, *TNNI3*, *TPM1*, *ACTC1*, *MYL2*, and *MYL3*. It is reported that other genes (*ACTN2*, *CSRP3*, *TNNC1*, *FHOD3*, *FLNC*, and *PLN*) contribute to approximately 5% of cases [150].

Of these, *MYBPC3* truncating variants have been found to be particularly associated with haploinsufficiency and a progressive clinical course [151].

From a therapeutic perspective, the range of pharmacologic options has expanded beyond traditional agents. Mavacamten, a selective myosin ATPase inhibitor, has demonstrated efficacy in reducing left ventricular outflow tract gradient and symptoms in obstructive HCM, and the consequences of this are especially significant for patients with sarcomeric mutations [148].

Gene therapy for HCM is still in the early stages of development.

TN-201, an AAV9-based vector carrying wild-type *MYBPC3*, is under investigation in the MyPEAK-1 trial (NCT05836259), targeting symptomatic non-obstructive HCM with confirmed *MYBPC3* mutations [152].

In animal models, AAV-mediated delivery of *MYBPC3* restored protein levels, prevented hypertrophy, and preserved contractile function [153].

Other experimental strategies include allele-specific RNA interference, which has delayed phenotype onset in *MYH6*-mutated mice [154]; exon-skipping antisense oligonucleotides, tested in *MYBPC3*-deficient mice to bypass frameshift mutations [155]; CRISPR/Cas9-mediated correction, demonstrated in human zygotes; and hiPSC-derived cardiomyocytes, although with debated results [156]. 

### 5.3. Cardiomyopathies with a Restrictive Phenotype

Restrictive cardiomyopathy (RCM) describes a functional phenotype characterized by impaired diastolic filling, normal or mildly increased wall thickness, and biatrial enlargement. Patients with RCM manifest typical signs and symptoms of HFpEF [2,18].

Infiltrative and storage diseases with known genetic etiology are a major cause of this phenotype and require targeted recognition. However, it can also occur in patients with DCM or HCM in the terminal stage [18].

Hereditary transthyretin amyloidosis (ATTRv), caused by autosomal dominant mutations in the *TTR* gene, leads to misfolding and myocardial deposition of transthyretin fibrils. Common mutations, such as Val122Ile and Thr60Ala, are associated with predominantly cardiac involvement. ATTRv typically presents in mid-to-late adulthood with progressive diastolic dysfunction, arrhythmias, and conduction disturbances [18]. Available therapies include tafamidis, a TTR stabilizer approved for cardiac ATTR, and gene-silencing agents such as patisiran, vutrisiran (siRNA), and inotersen (ASO), which reduce circulating mutant transthyretin and slow disease progression [107,108,110]. The subject of clinical studies is NTLA-2001, a CRISPR therapy that targets the *TTR* gene. Initial results suggest that it can effectively reduce serum TTR levels and is generally well tolerated [131].

Another genetic storage disease with restrictive features is Fabry disease, an X-linked lysosomal disorder caused by mutations in the *GLA* gene. The therapeutic approach involves the administration of enzyme replacement therapy (ERT) and migalastat, a pharmacologic chaperone.

The monogenic nature of Fabry disease makes it a suitable candidate for gene therapy. Heart-targeted AAV vectors, such as 4D-310 (INGLAXA trial, NCT04519749), have shown promising results in early-phase studies, with sustained enzyme activity and substrate reduction. However, some safety concerns, such as acute hemolytic uremic syndrome, have emerged, leading to a clinical hold [157,158]. 

Preliminary findings from the STAAR trial (NCT04046224), using a liver-directed AAV vector, also indicate potential clinical benefit of a systemic gene therapy approach [159]. 

A lentiviral stem cell-based approach (NCT03454893) was also tested, though discontinued [160].

A further example is Danon disease, caused by X-linked mutations in *LAMP2*, which typically affects males and presents early with massive biventricular hypertrophy, skeletal myopathy, and cognitive impairment. The cardiac phenotype often progresses rapidly toward restrictive dysfunction and heart failure [18]. Preclinical studies support the application of AAV9-mediated *LAMP2B* gene therapy, currently under early-phase investigation [115].

## 6. Conclusions

This review has explored the role of genetic variability in the efficacy of HF treatments. Despite the complexity of the genetic architecture underlying this heterogeneous syndrome, and the challenges involved in achieving consistent data, genetic variants, ranging from common SNPs to rare single-nucleotide variants, have been shown to influence drug metabolism, target receptor sensitivity, and susceptibility to adverse effects. Understanding these mechanisms is particularly relevant for optimizing pharmacological treatments. Furthermore, epigenetic modifications and gene–environment interactions modulate therapeutic response, reinforcing the rationale for a precision medicine approach in HF management.

Despite significant progress, several gaps remain. The clinical translation of pharmacogenomic findings is limited by the lack of large-scale, multi-ethnic validation studies and randomized controlled trials. While gene therapy strategies hold promise, they face challenges related to safety, delivery efficiency, long-term efficacy, and ethical considerations. In addition, epigenetic targets are not yet fully elucidated.

Nevertheless, the future of HF therapy will benefit from the development of a comprehensive, integrated precision medicine model that combines clinical, environmental, and genetic data to personalize treatment, improve outcomes, and minimize harm.

## Figures and Tables

**Table 1:** Summary of HF phenotypes, etiologies, and recommended therapies.

HF (LVEF)	Etiologies	Recommended Therapy
HFrEF (≤40%)	Ischemic heart disease: leading global cause, especially in high-income regions; associated with high mortality. Dilated/genetic cardiomyopathy: often familial, linked to *TTN*, *LMNA*, *FLNC* variants. HTN contributes to afterload stress and ventricular remodeling. Valvular disease (less frequent): especially post-ischemic mitral regurgitation. Myocarditis Chemotherapy-related cardiomyopathy Chagas disease	Class I: ARNI/ACEi, beta-blockers, MRAs, SGLT2i. Class I: Diuretics for congestion relief. ICD, CRT (Class I/IIa where indicated). Class IIa/IIb: Ivabradine, Vericiguat, IV iron, Digoxin (selected patients).
HFmrEF (41–49%)	Ischemic heart disease: intermediate prevalence. HTN: long-standing pressure overload; often with obesity and DM. Valvular heart disease: often degenerative. Genetic cardiomyopathy (less common). Myocarditis Aging population and AF frequently present.	Class I: SGLT2i, Diuretics for symptom relief. Class IIb: ARNI, ACEi/ARB, beta-blockers, MRAs (clinical judgement).
HFpEF (≥50%)	HTN: leads to stiff, non-compliant LV. Valvular/rheumatic disease ischemic heart disease: variable (24–52%). Obesity, DM, metabolic syndrome. Infiltrative cardiomyopathies (e.g., amyloidosis). AF and advanced age strongly associated.	Class I: SGLT2i, diuretics for volume overload, comorbidity management (HTN, DM, obesity, AF, OSAS).

**Abbreviations:** ACEi: Angiotensin-Converting Enzyme inhibitor; AF: Atrial Fibrillation; ARB: Angiotensin II Receptor Blocker; ARNI: Angiotensin Receptor–Neprilysin Inhibitor; CRT: Cardiac Resynchronization Therapy; DM: Diabetes Mellitus; HTN: Hypertension; ICD: Implantable Cardioverter Defibrillator; IV: Intravenous; MRA: Mineralocorticoid Receptor Antagonist; OSAS: Obstructive Sleep Apnea Syndrome; SGLT2i: Sodium Glucose Cotransporter-2 inhibitor.

**Table 2:** Gene Therapy Approaches in HF.

Transgene	Approach	Target	Status	Delivery	
TTR	siRNA (Patisiran, Vutrisiran)	Liver (*TTR* silencing)	Approved (FDA/EMA)	Lipid nanoparticles/GalNAc conjugation	[107,126]
TTR	ASO (Inotersen)	Liver (*TTR* silencing)	Approved (with monitoring)	Subcutaneous injection	[110]
miR-132	Anti-miR (CDR132L)	Heart (miR-132 silencing)	Phase 1b Clinical Trial	Intravenous injection	[111]
miR-199a-3p/miR-590-3p	miRNA mimics (e.g., miR-199a-3p)	Heart (regenerative miRNAs)	Preclinical (animal models)	Intramyocardial injection	[127]
MYBPC3, PKP2, LAMP2B	AAV-mediated gene replacement	Heart (loss-of-function mutations)	Clinical Trials (in progress)	AAV vectors (cardiotropic)	[114,128,129]
VEGF-A, IGF-1	modRNA (e.g., VEGF-A, IGF-1)	Heart (protein overexpression)	Phase 2 Clinical Trial (AZD8601)	Direct myocardial injection/nanoparticles	[120]
CaMKIIδ	CRISPR-Cas9 gene editing	Heart (pathogenic mutation correction)	Preclinical (animal models)	AAV or lipid nanoparticles	[130]
TTR	NTLA-2001 (CRISPR for TTR)	Liver (*TTR* gene editing)	Early Clinical Trial	Lipid nanoparticle infusion	[131]
SERCA2a	AAV1-mediated gene transfer	Cardiac calcium cycling	Phase 2 (CUPID trial failed)	Intracoronary AAV1	[132]
Microdystrophin	AAV-mediated gene replacement	Dystrophin restoration	Phase 1/2 (Duchenne cmp)	AAVrh74 (SRP-9001, PF-06939926)	[117,133]

**Table 3:** Therapeutic Implications of Different Genotypes in DCM.

Gene	Phenotype	Resp. to GDMT	Arrh. Risk	Therapeutic Implications
*TTN (TTNtv)* [141]	Mild/mod. DCM; recovery; acq. forms	Good	Mod.	Early GDMT; test in alc./PPCM/tox. DCM
*LMNA* [43]	Early-onset; CD (AVB, LBBB); rapid prog.	Poor	Very high	Early ICD; use LMNA Risk-VTA score
*FLNC* [141]	Fibrosis, VA; poor prog.	Limited	Very high	ICD if LGE on CMR; arrhyth. monitor
*RBM20* [140]	Young onset; malig. arrhyth.; aggressive	Limited	Very high	Early ICD; arrhyth. surveillance
*DSP* [139,142]	LDAC; epi. fibrosis; frequent PVCs	Variable	High	CMR; diff. from myocarditis; ICD if LGE
Others	CD, SND, atrial arrhyth.	Variable	Mod.	Personalized care; CD

**Abbreviations:** Resp.: Response; GDMT: Guideline-Directed Medical Therapy; Acq.: Acquired; PPCM: Peripartum Cardiomyopathy; CD: Conduction Disease; AVB: Atrioventricular Block; LBBB: Left Bundle Branch Block; VA: Ventricular Arrhythmias; Prog.: Progression; LGE: Late Gadolinium Enhancement; CMR: Cardiac Magnetic Resonance Imaging; Malig.: Malignant; LDAC: Left-Dominant Arrhythmogenic Cardiomyopathy; Epi.: Epicardial; PVCs: Premature Ventricular Contractions; Diff.: Differentiate; SND: Sinus Node Dysfunction.
